# NFATC2-mediated CST1 upregulation drives cholangiocarcinoma growth and metastasis

**DOI:** 10.1038/s41420-026-03036-8

**Published:** 2026-03-25

**Authors:** Wei Zhao, Jing Zhao, Kun Li, Jian Shi, Liyuan Cong, Guangyi Yu

**Affiliations:** 1 Department of Hepatobiliary and Pancreatic Surgery, The Affiliated Hospital of Qingdao University, Qingdao, PR China; 2 Department of Pathology, The Affiliated Hospital of Qingdao University, Qingdao, PR China

**Keywords:** Metabolomics, Oncogenesis

## Abstract

Intrahepatic cholangiocarcinoma (CCA) is a highly aggressive malignancy arising from the intrahepatic biliary epithelium with insidious onset and dismal clinical outcomes. The lack of reliable early diagnostic markers and effective therapeutic targets underscores the urgent need for novel intervention strategies. Integrated evaluation of public transcriptomic datasets and local validation cohort with survival analysis were performed to assess expression pattern and prognostic significance of cystatin SN (CST1) in CCA. Functional characterization was performed via gain- and loss-of-function experiments in HuCCT1 and RBE cells, complemented by murine orthotopic liver implantation and pulmonary metastasis models. We found that CST1 was significantly upregulated in human CCA tissues. Elevated CST1 expression predicted unfavorable prognosis in CCA patients. Subsequently functional studies revealed that overexpression of CST1 suppressed cellular senescence markers, as evidenced by decreased senescence-associated β-galactosidase activity and downregulated senescence-associated secretory phenotype factors (IL-6, CCL20). Concomitantly, CST1 overexpression enhanced cell proliferation, migration, invasion, and in vivo metastatic capacity. Integrated multi-omics profiling identified CST1-mediated suppression of pyrimidine metabolism through TYMS downregulation. However, exogenous thymidine supplementation failed to rescue proliferation defects upon CST1 knockdown, indicating that CST1-promoted tumor growth is independent of pyrimidine metabolism. Mechanistically, NFATC2 transcriptionally activates CST1, which subsequently abrogates senescence through SOX4 stabilization; ectopic SOX4 expression rescues senescence induced by CST1 depletion. These findings establish CST1 as a promising therapeutic target and provide mechanistic insights for CCA intervention strategies.

## Introduction

Intrahepatic cholangiocarcinoma (CCA) represents 5–10% of primary liver malignancies, with a globally increasing incidence trend documented in recent decades [1]. Characterized by insidious onset and absence of early-specific symptoms, CCA exhibits highly invasive growth patterns and intrinsic resistance to conventional chemotherapy, thereby contributing to its dismal prognosis. CCA accounts for approximately 2% of all cancer-related deaths worldwide annually, underscoring the urgent need to elucidate its molecular pathogenic mechanisms for the development of targeted therapeutic strategies.

Cellular senescence is an important biological process in which cells enter an irreversible state of growth arrest. During successive cell divisions, DNA replication-induced telomere shortening eventually triggers the activation of the DNA damage response, leading to cell cycle arrest [2, 3]. Beyond physiological aging, cells can undergo premature senescence in response to diverse stressors, such as oxidative damage, radiation exposure, and the effects of chemotherapy drugs [4]. Senescent cells exhibit several characteristic features: loss of proliferative capacity, activation of tumor suppressor signaling pathways, changes in cell morphology, increased senescence-associated β-galactosidase (SA-β-Gal) activity, and a transformed secretory phenotype (e.g., release of pro-inflammatory cytokines). It is worth noting that malignant tumor cells often bypass senescence through genetic alterations, thereby achieving unlimited proliferation [5]. From this perspective, cell senescence constitutes an important defense barrier for the body against tumor development.

Cysteine proteases, such as cathepsins and papain-like enzymes, are ubiquitously expressed proteases involved in critical biological processes ranging from bone remodeling and immune responses to cancer progression [6]. To counterbalance their activity, the cystatin superfamily serves as an endogenous regulator of cysteine protease function. Among these, Cystatin SN (CST1), a secreted type 2 cystatin, is frequently overexpressed in multiple malignancies [7]. Emerging evidence implicates CST1 in driving tumor development and metastatic spread across various cancers, including gastrointestinal, breast, thyroid, lung, and liver malignancies [8–13]. Notably, CST1 depletion has been shown to trigger cellular senescence in breast and colorectal cancers [14]. However, its biological significance in CCA remains largely unexplored.

In this study, we sought to (a) elucidate the functional role of CST1 in regulating CCA cell proliferation, migration, invasion, senescence, and in vivo tumor growth/metastasis; (b) identify the upstream regulatory mechanisms driving CST1 overexpression in CCA; and (c) define CST1-regulated molecular networks through integrated proteomic and metabolomic profiling.

## Results

### CST1 is upregulated gene in CCA and associated with poor prognosis

**Fig. 1:**
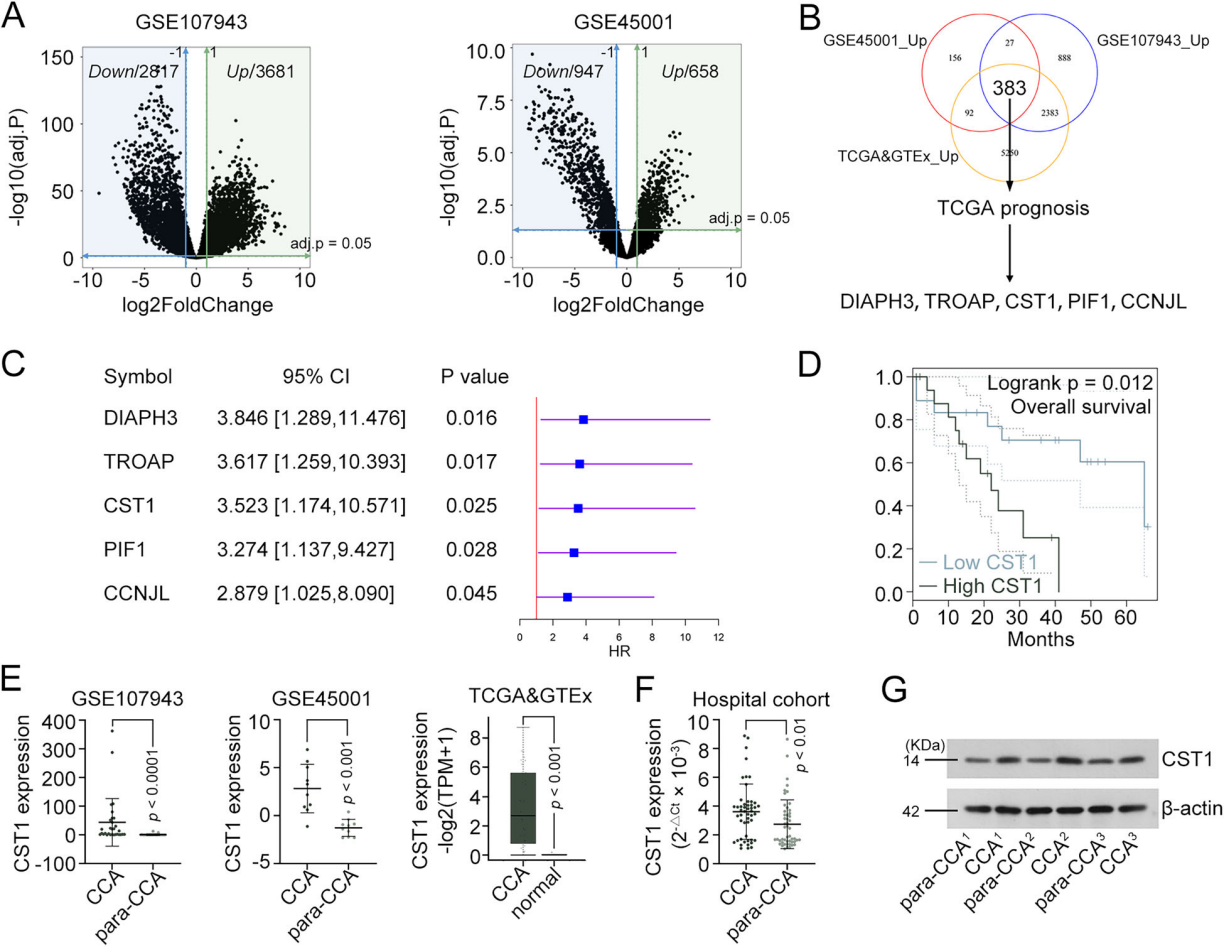
CST1 is upregulated gene in CCA and associated with poor prognosis. **A** Volcano plot of DEGs in GSE107943 (30 CCA/27 normal tissues) and GSE45001 (10 CCA/10 normal tissues) datasets, generated using thresholds of |log2FC | ≥ 1, adjusted p-value < 0.05. **B** Venn diagram depicting consistently upregulated genes among GSE107943, GSE45001 and TCGA_GTEx. **C** Cox proportional hazards regression analysis was performed via R package to assess the impact of gene expression on the survival of patients. **D** Survival curves of CCA patients stratified by CST1 expression levels (high vs. low). **E** CST1 mRNA expression levels in CCA and normal tissues across TCGA&GTEx integration (36 CCA/9 normal), GSE107943, and GSE45001 datasets. **F** CST1 mRNA expression levels in CCA and normal tissues from a local hospital cohort (50 CCA/50 normal). **G** Three pairs of tissues were randomly selected from the local cohort for western blot analysis of CST1 protein expression. Data in (**F**) were presented as mean ± SD. DEGs differentially expressed genes; FC fold change. Full and uncropped western blots were shown in Supplemental Material.

Transcriptomic analysis of GSE107943 and GSE45001 datasets under stringent criteria (|log2FC | ≥ 1, adjusted *p*-value < 0.05) identified 6498 DEGs (3691 upregulated) and 1605 DEGs (658 upregulated), respectively (Fig. 1A). Intersection analysis across these datasets and TCGA_GTEx revealed 383 consistently upregulated genes (Fig. 1B). Cox proportional hazards regression analysis further pinpointed five candidates—DIAPH3, TROAP, CST1, PIF1, and CCNJ1—as significantly associated with patient survival (*p* < 0.05), all exhibiting hazard ratios > 1 indicative of poor prognosis (Fig. 1B, C). Among these, CST1 emerged as an oncogene with unknown functions in CCA. TCGA data showed that elevated CST1 expression correlated with reduced overall survival (Fig. 1D) and was consistently upregulated across all three datasets (Fig. 1E). These bioinformatics findings were subsequently validated in local clinical specimens, which demonstrated markedly elevated CST1 expression at both mRNA and protein levels (Fig. 1F, G).

### CST1 knockdown suppresses proliferation and metastasis in CCA cells

**Fig. 2:**
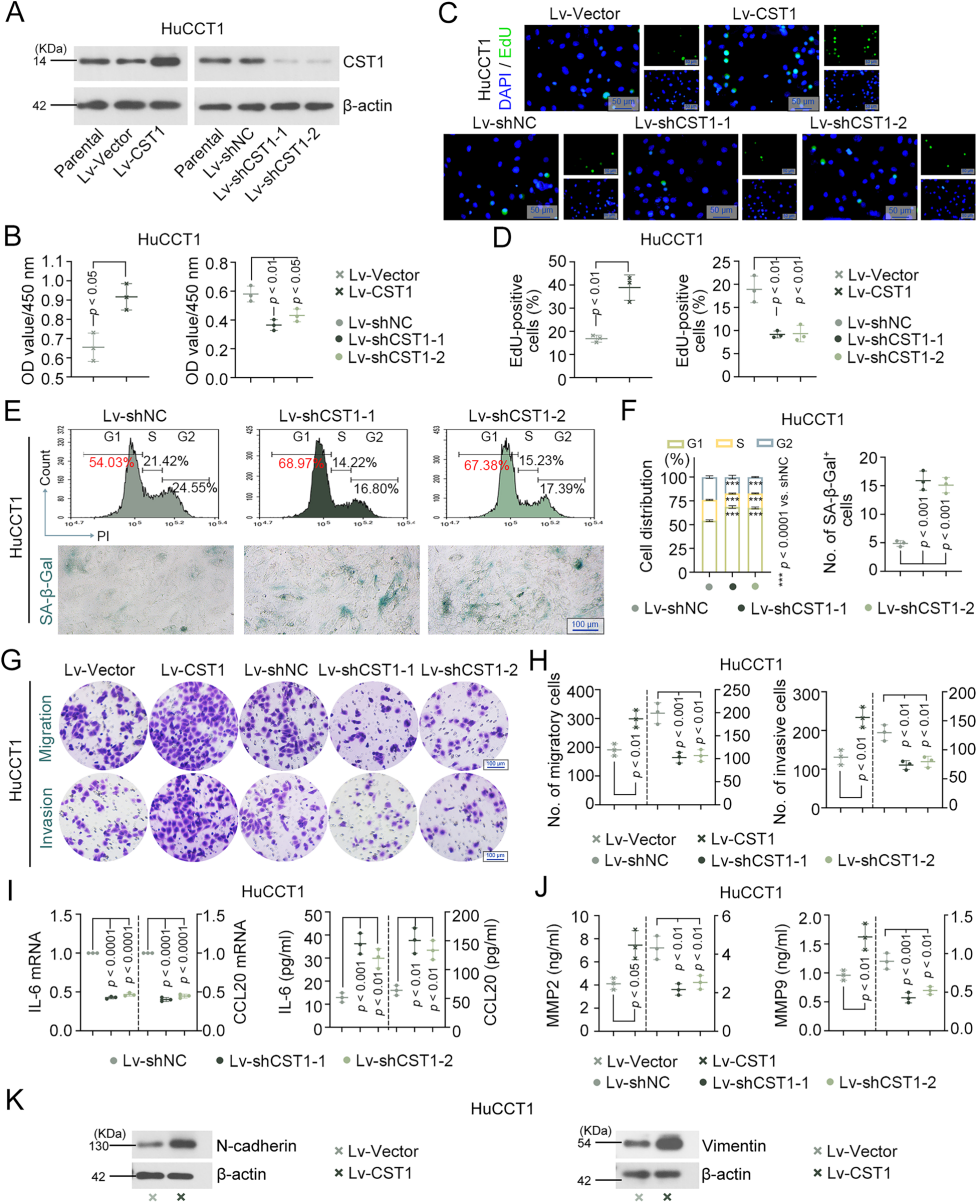
CST1 knockdown suppresses proliferation and metastasis in HuCCT1 cells. **A** Expression of CST1 in human HuCCT1 cells was detected by western blot. **B** The number of viable cells after 48 h of incubation was evaluated by the CCK8 assay. **C**, **D** The DNA replication capacity of HuCCT1 cells was assessed and quantified by EdU incorporation assay (C, bar = 50 μm), with EdU-positive cells subsequently quantified (**D**). **E**, **F** Cell cycle distribution of HuCCT1 cells was analyzed by flow cytometry (**E**: upper; **F**: left). Cellular senescence in HuCCT1 cells was assessed by SA-β-gal staining (bar = 100 μm), with positive cells subsequently quantified (E: lower; F: right). **G**, **H** Migration and invasion capabilities of HuCCT1 cells were evaluated by Transwell chamber assays with and without Matrigel (bar = 100 μm). **I** IL-6, and CCL20 mRNA expression and secreted levels in HuCCT1 cells were assessed by RT-qPCR and ELISA, respectively. **J** Secretion levels of invasion-related factors MMP2 and MMP9 in HuCCT1 cell supernatants were quantified by ELISA. **K** Expression of mesenchymal markers N-cadherin and Vimentin in HuCCT1 cells were detected by western blot. Data in (**B**, **D**, **F**, **H–J**) were presented as mean ± SD. SA-β-gal, senescence-associated β-galactosidase. Full and uncropped western blots are shown in Supplemental Material.

To investigate the function of CST1 in CCA, lentivirus-mediated overexpression and knockdown systems were used to upregulate and downregulate CST1 expression in two CCA cell lines, HuCCT1 and RBE, respectively (Fig. 2A and S1A). CCK8 assay data showed that after 48 h of incubation, the number of viable cells in the CST1 overexpression group increased significantly, while that in the CST1 silencing group decreased (Fig. 2B and S1B). EdU incorporation assays further confirmed that CST1 overexpression effectively promoted tumor cell DNA replication, as evidenced by increased EdU-positive cells, whereas CST1 silencing inhibited cellular DNA synthesis (Fig. 2C, D and S1C, D). These results collectively indicated that CST1 silencing effectively suppressed tumor cell proliferation. Additionally, flow cytometry, senescence β-galactosidase staining kit, RT-qPCR, and ELISA were used to evaluate the regulatory effect of CST1 on the senescence status of tumor cells. Flow cytometry analysis revealed that CST1 silencing arrested tumor cells in the G1 phase (Fig. 2E, F and S1E, F). Results from the β-galactosidase kit assay showed that CST1 silencing increased the proportion of SA-β-gal-positive senescent cells (Fig. 2E, F and S1E, F). RT-qPCR and ELISA jointly confirmed that CST1 silencing reduced the expression levels of senescence-associated factors IL-6 and CCL20 in both cells and cell supernatants (Fig. 2I and S1I). These findings suggested that CST1 silencing inhibited tumor cell proliferation and induced tumor cell senescence.

Transwell chamber assays, with and without Matrigel, were used to evaluate tumor cell migration and invasion. Results showed that CST1 overexpression significantly promoted cell migration to the lower surface of the microporous membrane, whereas CST1 silencing significantly inhibited migration (Fig. 2G, H and S1G, H). In Matrigel-coated invasion assays, CST1 overexpression enhanced the ability of tumor cells to penetrate Matrigel, while CST1 silencing suppressed cell invasion (Fig. 2G, H and S1G, H). Matrix metalloproteinases MMP2 and MMP9 mediate tumor migration and invasion by degrading the extracellular matrix (ECM) [15]. ELISA analysis revealed that the contents of MMP2 and MMP9 in cell supernatants were increased after CST1 overexpression, yet decreased after CST1 silencing (Fig. 2J and S1J). Western blot results showed that CST1 overexpression upregulated the expression of mesenchymal markers N-cadherin and Vimentin, suggesting that CST1 overexpression induced the epithelial-mesenchymal transition (EMT) process in CCA cells (Fig. 2K and S1K).

### CST1 depletion suppresses CCA growth and metastasis in vivo

**Fig. 3:**
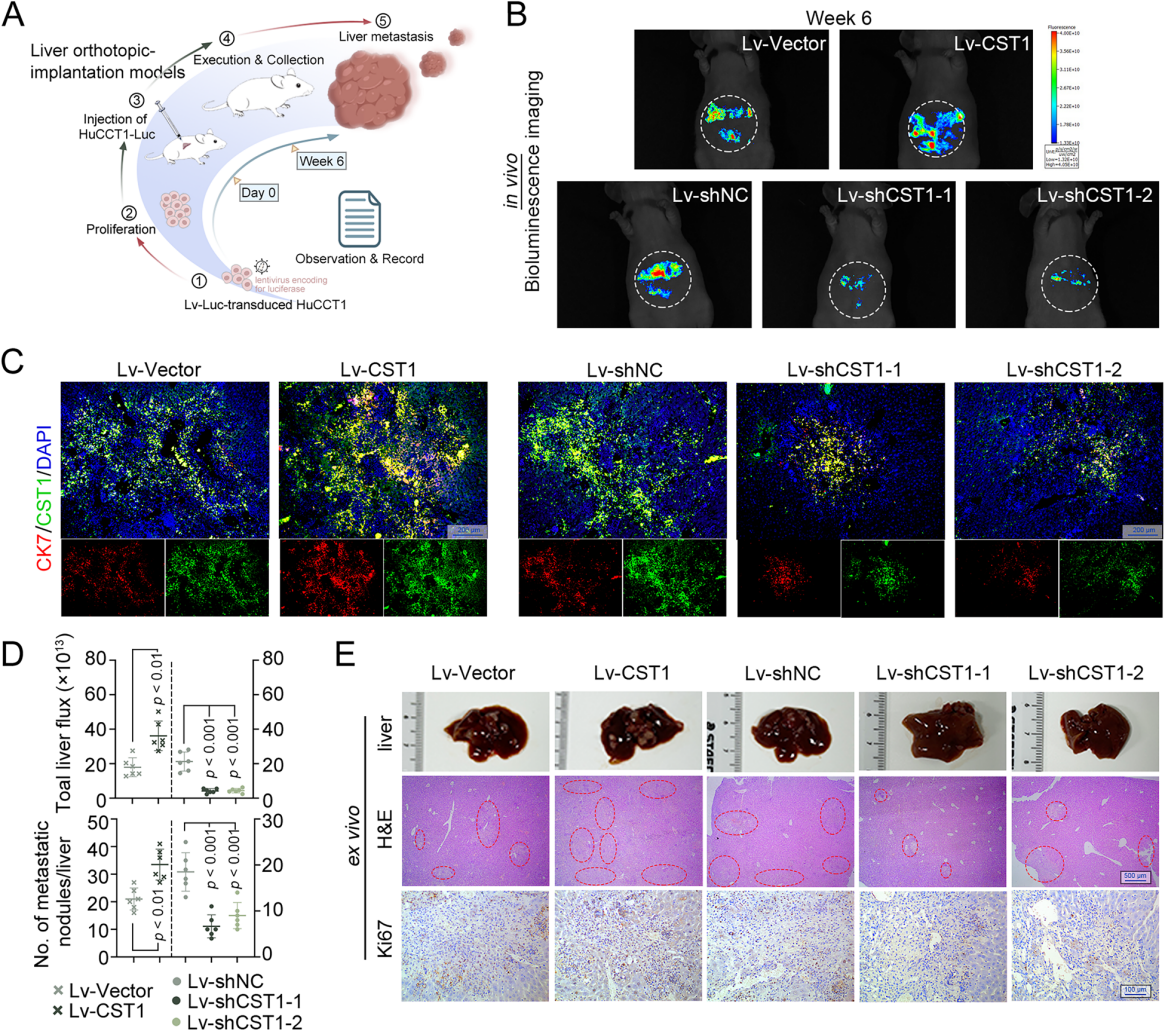
CST1 silencing suppresses CCA growth and metastasis in vivo. **A** Schematic illustration of orthotopic liver implantation models inoculated with HuCCT1 cells. **B** Representative bioluminescent images of liver lesions. **C** Representative double immunofluorescence images evaluating CCA progression (bar = 200 μm). **D** Quantification of photon flux (upper) and intrahepatic nodule numbers (lower). **E** Representative gross morphology of liver tumors (upper), H&E staining (middle), and Ki67 immunohistochemistry (lower). Data in (**D**) were presented as mean ± SD.

Bioluminescence imaging revealed that CST1 overexpression significantly enhanced tumor burden, evidenced by increased photon flux in the orthotopic liver xenografts (Fig. 3A, B, D). Double immunofluorescence staining confirmed elevated expression of the biliary marker cytokeratin 7 (Fig. 3C). Histologically, H&E staining and morphometric analysis demonstrated that CST1 overexpression increased the number of intrahepatic nodules, while IHC analysis of Ki67 indicated accelerated tumor cell proliferation in the CST1 overexpression group. By contrast, CST1 knockdown attenuated tumor growth (Fig. 3D, E).

In the lung metastasis model, CST1 overexpression similarly promoted metastatic colonization, as indicated by increased metastatic foci (Fig. S2A, B), while CST1 silencing effectively suppressed pulmonary metastasis (Fig. S2C).

### Proteomics and metabolomics analyses of differential proteins and metabolites

**Fig. 4:**
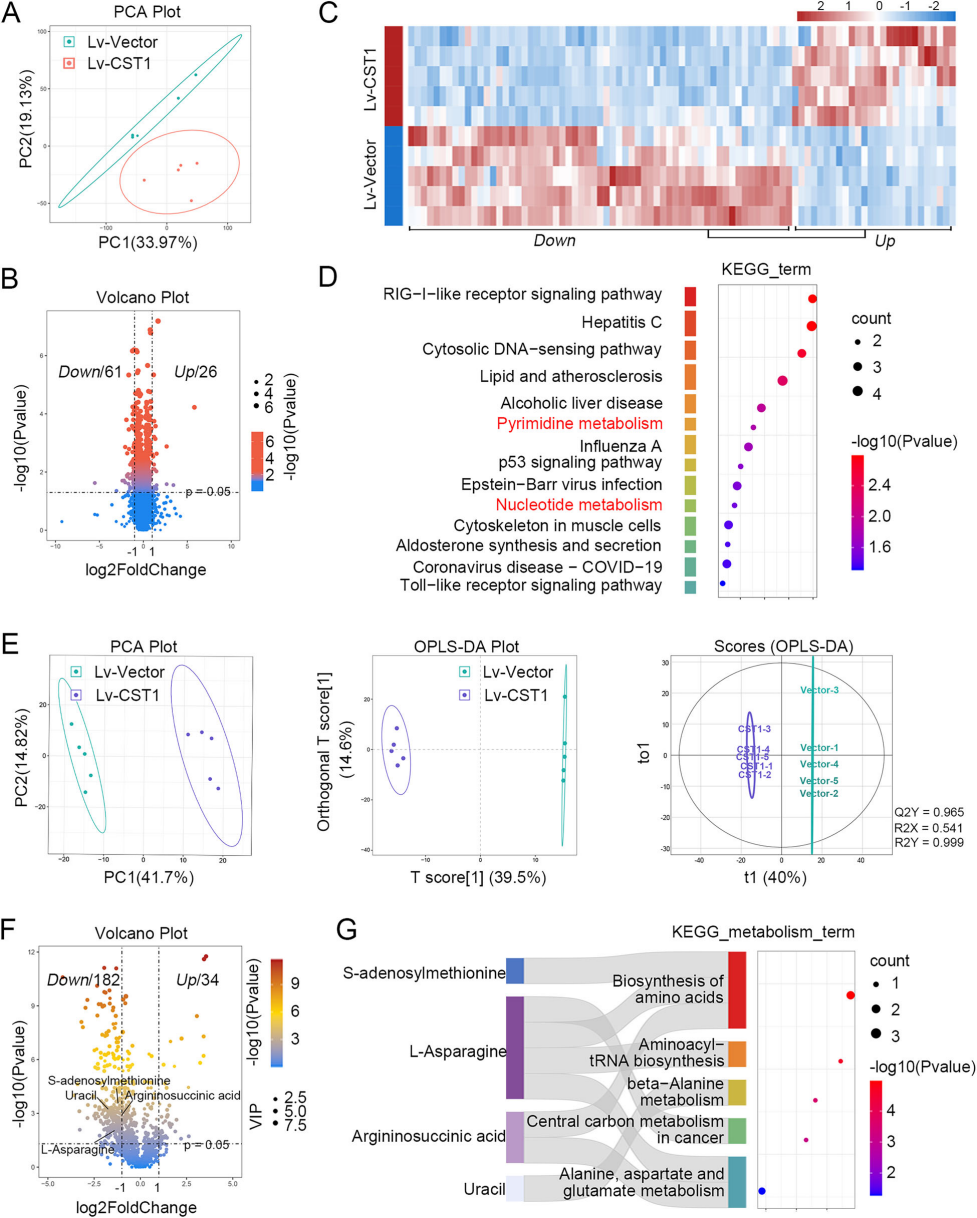
Proteomics and metabolomics analyses of differential proteins and metabolites. **A** PCA plot derived from proteomics profiles comparing between CST1 overexpressing cells and control cells. Volcano **B** and heatmaps **C** of DEPs in HuCCT1 cells with CST1 overexpression and their control counterparts. **D** KEGG functional enrichment analysis for DEPs. **E** PCA plot, OPLS-DA plot, and OPLS-DA score scatter plot derived from metabolomics profiles comparing between CST1 overexpressing cells and control cells. **F** Volcano of DEMs in HuCCT1 cells with CST1 overexpression and their control counterparts. **G** KEGG functional enrichment analysis for DEMs. PCA principal component analysis, OPLS-DA orthogonal projections to latent structures-discriminant analysis, DEPs differentially expressed proteins, DEMs differentially expressed metabolites, KEGG Kyoto encyclopedia of genes and genomes.

Label-free proteomics was employed to characterize DEPs in HuCCT1 cells with CST1 overexpression and their control counterparts. PCA results showed that there were significant differences between CST1 overexpressing cells and control cells (Fig. 4A). A volcano plot identified 61 downregulated and 26 upregulated proteins using the criteria of |Log₂FC | > 1 and *p*-value < 0.05 (Fig. 4B). A heatmap visually represented the expression patterns of these DEPs (Fig. 4C). KEGG pathway enrichment analysis highlighted significant enrichment in tumor metabolic pathways, particularly pyrimidine metabolism (Fig. 4D). Given the critical role of pyrimidine metabolism in nucleic acid synthesis and cell proliferation [16–19], we conducted metabolomics analysis to further investigate metabolic perturbations.

Metabolomics data quality assessment showed excellent aggregation of quality control samples in both PCA and OPLS-DA models (Fig. 4E), validating data reliability. Using stringent criteria of |Log₂FC | > 1, *p*-value < 0.05 and VIP > 1, a total of 216 significantly DEMs were identified (Fig. 4F). KEGG pathway analysis revealed predominant involvement in central carbon metabolism in cancer, β-alanine metabolism, aminoacyl-tRNA biosynthesis, and amino acid biosynthesis (Fig. 4G). Key metabolites including uracil, argininosuccinic acid (ASA), L-asparagine (Asn), and S-adenosylmethionine (SAM) were prominently enriched in these pathways, suggesting their potential as metabolic effectors of CST1 function (Fig. 4G).

### Integrated analysis reveals the core metabolic network regulated by CST1

**Fig. 5:**
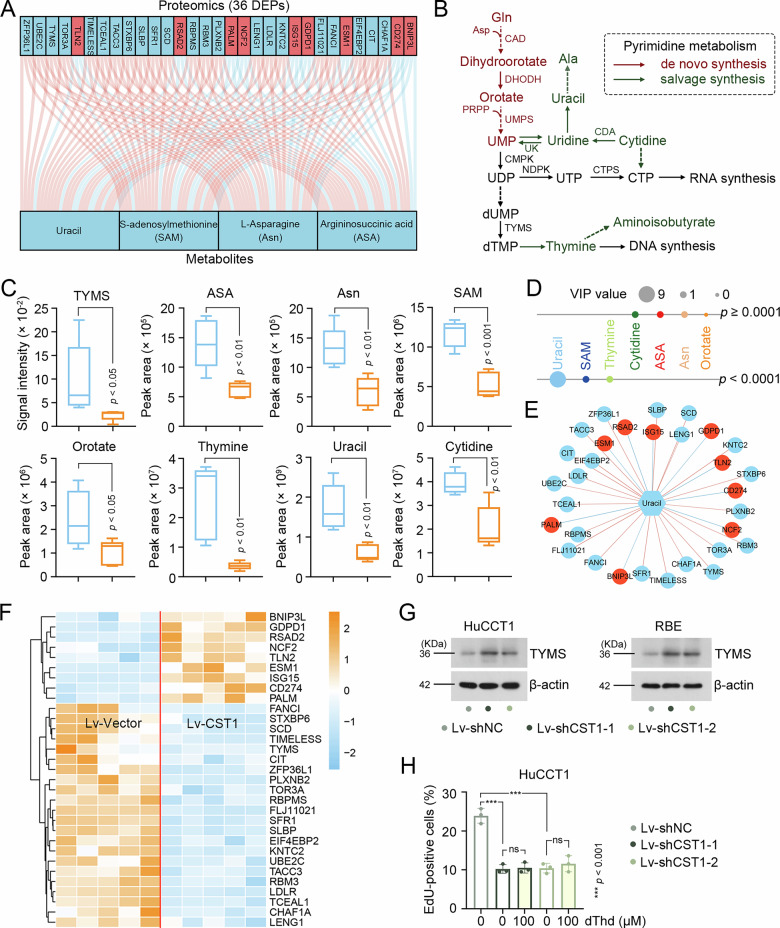
Integrated analysis reveals the core metabolic network regulated by CST1. **A** Sankey diagram depicting the correlations between proteins and key metabolites regulated by CST1. Red lines represent positive correlation, and blue lines represent negative correlation. Red grid indicates up-regulation and blue grid indicates down-regulation in HuCCT1 cells with CST1 overexpression. **B** Schematic representation of pyrimidine metabolism. **C** The peak area of metabolites in HuCCT1 cells. **D** The potential contribution of DEMs that responded to CST1 overexpression. **E** Network analysis of protein-metabolite interactions. **F** Heatmaps of DEPs correlated to uracil in HuCCT1 cells. DEPs, differentially expressed proteins; DEMs, differentially expressed metabolites. **G** Protein expression of TYMS in HuCCT1 cells were detected by western blot. **H** To evaluate pyrimidine dependency, CST1-depleted HuCCT1 cells were treated with 100 µM dThd for 24 h prior to EdU incorporation assay. Data in (**C**, **H**) were presented as mean ± SD. Ala alanine, Asp aspartic acid, CAD carbamoyl-phosphate synthetase 2 [1], aspartate transcarbamylase [2], and dihydroorotase [3], CDA cytidine deaminase, CMPK cytidine/uridine monophosphate kinase, CTP cytidine triphosphate, CTPS cytidine triphosphate synthase, DHODH dihydroorotate dehydrogenase, Gln glutamine, NDPK nucleoside diphosphate kinase, PRPP phosphoribosyl pyrophosphate, UK uridine kinase UMP uridine monophosphate, UMPS uridine monophosphate synthase, UDP uridine diphosphate, UTP uridine triphosphate, dUMP deoxyuridine monophosphate, dTMP deoxythymidine monophosphate, TYMS thymidylate synthase, dThd thymidine. Full and uncropped western blots were shown in Supplemental Material.

Metabolomic analysis showed that uracil, a fundamental building block of RNA, maintains homeostasis through coordinated de novo synthesis and salvage pathways that sustain the dynamic equilibrium of the intracellular nucleotide pool. Notably, the concentration changes of ASA, Asn, and SAM—key precursor molecules in the de novo pyrimidine synthesis pathway—further corroborated the regulatory role of CST1 in nucleotide metabolism [16]. By integrating proteomic and metabolomic datasets, we identified 36 DEPs exhibiting significant correlations (|Pearson R | > 0.6) with the four metabolites (Fig. 5A). Among these, thymidylate synthase (TYMS), a rate-limiting enzyme in pyrimidine metabolism, plays a central role in catalyzing the methylation of deoxyuridine monophosphate (dUMP) to deoxythymidine monophosphate (dTMP), a critical step in DNA synthesis (Fig. 5A, B). Metabolomic data showed that CST1 overexpression significantly downregulated the abundance of metabolites in the pyrimidine synthesis/degradation pathway (Fig. 5C), with uracil displaying the highest variable importance in the projection (VIP > 9), indicating its predominant contribution to intergroup differences (Fig. 5D). Network analysis further visualized the strong correlation between uracil levels and these DEPs (Fig. 5E), while the hierarchical clustering heatmap systematically revealed the co-expression patterns of these proteins in CST1-overexpressing and control groups (Fig. 5F). However, thymidine rescue assays indicated that proliferation defects induced by CST1 silencing could not be reversed by exogenous thymidine supplementation (Fig. 5G, H).

### Gender determining region Y-box 4 (SOX4) mediates CST1-driven proliferation and senescence evasion in CCA

**Fig. 6:**
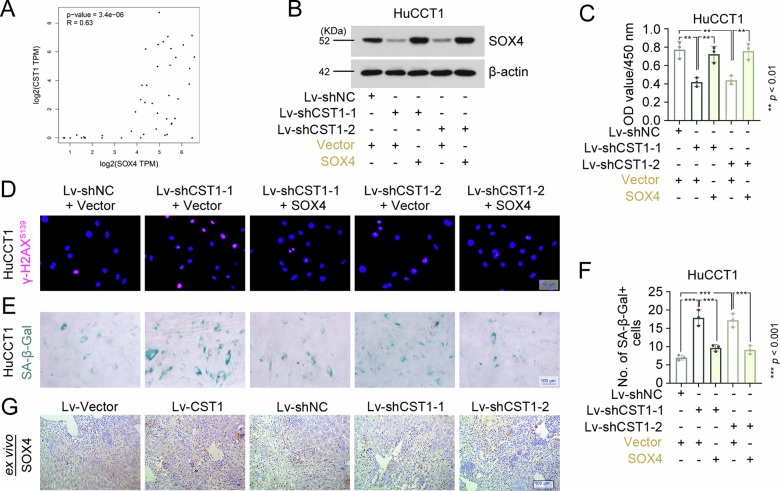
SOX4 mediates CST1-driven proliferation and senescence evasion in CCA. **A** GEPIA analysis of the correlation between CST1 and SOX4 expression in CCA. **B** SOX4 protein levels in HuCCT1 cells assessed by western blot. **C** Cell viability measured by CCK-8 assay at 48 h post-treatment. **D** Immunofluorescence staining for the DNA damage marker γ-H2AX (scale bar, 50 μm). **E**, **F** Cellular senescence evaluated by SA-β-gal staining (E; bar, 100 μm) and quantification of positive cells (**F**). **G** Representative immunohistochemical staining for SOX4 in liver tumors (bar, 100 μm). Data in (**C**, **F**) were presented as mean ± SD. Full and uncropped western blots were shown in Supplemental Material.

Building upon our observation that CST1 silencing induces proliferative arrest and senescence (Fig. 2, S1), we investigated the underlying mechanisms through integrative proteomic analysis (Fig. 4D). This approach identified SOX4—an established regulator of cellular senescence in cancer [20]—as a downstream effector positively regulated by CST1. We therefore hypothesized that CST1 exerts its oncogenic effects by modulating SOX4. Bioinformatics analysis of the GEPIA database revealed significant positive correlation between CST1 and SOX4 expression in CCA (Fig. 6A), which was corroborated at the protein level by our proteomic data showing CST1-driven SOX4 upregulation (Fig. 4C). Conversely, CST1 silencing reduced SOX4 expression in HuCCT1 cells, whereas ectopic SOX4 reintroduction restored its abundance in CST1-silenced HuCCT1 cells (Fig. 6B). Functional rescue experiments demonstrated that SOX4 overexpression in CST1-silenced cells restored cell viability (Fig. 6C), diminished DNA damage (Fig. 6D), and decreased SA-β-Gal-positive senescent populations (Fig. 6E, F). The liver orthotopic xenografts exhibited concordant upregulation of CST1 and SOX4 (Fig. 6G).

### NFATC2 activates CST1 transcription

To investigate the upstream transcriptional regulation of CST1, we analyzed the transcriptomic dataset GSE101323 (melanoma) and identified nuclear factor of activated T cells 2 (NFATC2) as a positive regulator—NFATC2 knockdown reduced CST1 expression by 3.87-fold (*p* < 0.05). Bioinformatics analysis using JASPAR revealed putative NFATC2 binding motifs within the CST1 promoter (Fig. S3A). We next experimentally validated this regulatory axis in HuCCT1 cells. RT-qPCR and Western blot confirmed efficient NFATC2 overexpression and knockdown (Fig. S3B–D), which respectively upregulated and downregulated CST1 at both mRNA and protein levels (Fig. S3B–D). Mechanistic investigation demonstrated that NFATC2 activates CST1 transcription. Dual-luciferase assays in 293 T cells demonstrated NFATC2-mediated activation of CST1 promoter, and the -2000/-1000 region was dispensable for this activation (Fig. S3E), suggesting critical NFATC2 response elements were located outside the -2000/-1000 region. ChIP-PCR assays, using primers spanning predicted binding sites in regions 3-5, confirmed NFATC2 occupancy at specific promoter regions (Fig. S3F). Further ChIP-PCR analysis validated NFATC2 recruitment to all three binding sites, as evidenced by specific amplification in NFATC2-immunoprecipitated DNA compared with input and IgG controls (Fig. S3G). Finally, EMSA confirmed direct physical interaction between NFATC2 and the CST1 promoter (Fig. S3H). Using purified NFATC2 protein and biotinylated oligonucleotides spanning region 3, we observed specific DNA-protein complex formation (lane 2), which was competitively inhibited by excess unlabeled wild-type probes (lane 6) but not by mutant oligonucleotides (lane 4), establishing sequence-specific binding of NFATC2 to region 3 (Fig. S3H).

### NFATC2 functions as an upstream regulator of CST1

Given our prior evidence that NFATC2 drives CCA proliferation and metastasis [21], we investigated whether NFATC2 functions as an upstream regulator of CST1. We discovered that NFATC2 additionally suppressed cellular senescence—a previously uncharacterized function—as evidenced by the induction of senescence markers upon NFATC2 knockdown in control cells (shNC+Lv-vector versus shNFATC2+Lv-vector; Fig. S4A–F). To determine whether NFATC2 mediates CST1-driven oncogenesis, we performed epistasis analysis by knocking down NFATC2 in CST1-overexpressing HuCCT1 cells. Notably, NFATC2 knockdown weakened CST1-mediated oncogenic effects, as demonstrated by decreased cell viability (Fig. S4A), blockage of G1-S phase cell cycle progression (Fig. S4B, D), increased SA-β-gal positive senescent cells (Fig. S4C, E), and suppressed migration and invasion capacities (Fig. S4F–H). These findings establish NFATC2 as a critical regulator of CST1’s proliferative, anti-senescent, and metastatic functions in CCA.

## Discussion

Through integrated analysis of public transcriptomic datasets (GSE107943, GSE45001, TCGA&GTEx) and a local validation cohort (*n* = 50), CST1 was identified as a significantly upregulated gene in CCA. CCA patients with high CST1 expression exhibited a worse 5-year overall survival. Consistent with its established oncogenic roles in breast, lung, gastric, liver and colorectal cancers, this study demonstrates that high CST1 expression promotes CCA growth and metastasis through both in vitro and in vivo experiments. Our findings highlight CST1’s potential as a prognostic biomarker and therapeutic target for CCA.

CST1, a canonical cysteine protease inhibitor with high specificity for cathepsin B (CTSB), L, and S, exhibits paradoxical pro-tumorigenic effects despite its inhibitory function. While cathepsins are well-established promoters of tumor invasion through ECM degradation at invasive fronts, and their pharmacological inhibition suppresses malignancy, CST1 paradoxically demonstrates similar pro-tumorigenic properties [22–25]. Recent mechanistic studies have reconciled this apparent contradiction by revealing that CST1 functions as a context-dependent modulator rather than a simple suppressor of proteolytic activity. Specifically, CST1 antagonizes cystatin C-mediated CTSB inhibition in cancer cells, thereby maintaining optimal protease activity required for cancer cell fitness [8]. Additionally, CST1 knockdown decreases extracellular CTSB activity, leading to cellular senescence through GSK3β Ser9 phosphorylation-mediated activation of glycogen synthase and subsequent glycogen accumulation [14]. Beyond its regulatory role in proteolysis, CST1 exerts pleiotropic oncogenic effects through cathepsin-independent mechanisms. Silencing of CST1 inhibits glycolysis and triggers autophagy-dependent ferroptosis [9, 26], while impairing PI3K-AKT, MAPK, and NF-κB p65 phosphorylation [27–29]. Furthermore, CST1 knockdown reverses EMT progression, highlighting its essential role in pro-metastatic potential [12]. These findings position CST1 as a unique regulatory node that fine-tunes proteolytic activity while simultaneously activating multiple oncogenic pathways, thereby explaining its paradoxical promotion of tumor progression despite its classification as a protease inhibitor.

Proteomic analysis identified 87 CST1-regulated proteins in CCA, with KEGG enrichment analysis showing significant overrepresentation in pyrimidine metabolism. This metabolic pathway constitutes a complex enzymatic network integrating the salvage of free nucleosides and bases, de novo synthesis from amino acids and ribose precursors, and the catalytic degradation of pyrimidines. It generates the nucleotide pool required for cell proliferation along with purine metabolism. Pyrimidine metabolism-related genes have been shown to drive tumorigenesis and metastasis, as well as confer chemotherapy resistance in CCA [30, 31]. Notably, proteomic data revealed that CST1 overexpression significantly downregulated TYMS in CCA cells (Fig. 6F). Integrative metabolomic analysis further confirmed reduced levels of key pyrimidine metabolism intermediates, including uracil and thymine, indicating CST1-mediated suppression of pyrimidine metabolism in CCA. However, despite CST1’s clear impact on pyrimidine metabolism and TYMS expression, proliferation defects induced by CST1 silencing could not be reversed by exogenous thymidine supplementation (Fig. 5H). This metabolic rescue failure suggests that CST1-driven tumor proliferation is independent of pyrimidine metabolism-mediated nucleotide synthesis, or that CST1 regulates proliferation through TYMS-independent mechanisms.

Given the dissociation between metabolic effects and proliferative rescue, we investigated whether CST1 suppresses cellular senescence—a stable growth-arrest state that constitutes a barrier to tumorigenesis. Here, we observed that CST1 knockdown induced senescence in both p53-mutant HuCCT1 and p53-wild-type RBE cells, indicating that this phenotype is independent of p53 status. Furthermore, CST1 overexpression did not significantly alter p16INK4a (CDKN2A) protein levels, suggesting that CST1 regulates senescence through mechanisms distinct from the canonical p53/p21 and p16INK4a/Rb pathways. Mechanistically, we identified SOX4—a known senescence suppressor [20]—as a direct downstream target of CST1. Ectopic SOX4 expression rescued cells from CST1 knockdown-induced senescence, establishing the CST1-SOX4 axis as a regulator of cellular senescence. Elucidation of the specific molecular mechanisms by which CST1/SOX4 modulate senescence awaits further investigation.

Several transcription factors, including HOXC10 and TFAP2A, have been identified as regulators of CST1 gene expression in cancer cells [32, 33]. TFAP2A, highly expressed in CCA, promotes EMT progress in CCA cells [34], yet its role in promoting CCA cell proliferation or inhibiting senescence remains uncharacterized. TCGA-CCA analysis showed no significant difference in HOXC10 expression between CCA and normal tissues (*p*-value > 0.05), contrasting with its reported role in other malignancies [32, 35, 36]. This study identifies NFATC2 as a novel transcription factor that upregulates CST1 mRNA expression, mediates CST1-driven proliferation, migration, and invasion, and suppresses cellular senescence. These findings establish NFATC2 as a key regulator of the CST1 oncogenic axis in CCA.

This study has several limitations that warrant acknowledgment. A comprehensive clinicopathological correlation analysis is needed to evaluate the association between CST1 expression levels and standard clinical parameters, such as patient age, tumor size, and histological grade. However, the current cohort size limits our ability to perform statistically meaningful assessments of these relationships. Future studies will aim to incorporate these critical preclinical investigations to further validate our findings and elucidate the functional mechanisms of CST1 in CCA progression.

In summary, our findings identify a novel NFATC2-CST1-SOX4 regulatory axis that drives CCA progression through senescence evasion, proliferation and metastatic dissemination. By demonstrating that transcriptional upregulation of CST1 by NFATC2 promotes oncogenic signaling via SOX4, we establish CST1 as a critical molecular node in CCA pathogenesis. These findings position CST1 as a promising therapeutic target for intrahepatic CCA intervention, warranting further investigation into its translational potential.

## Materials and methods

### Data acquisition and analysis

The Gene Expression Omnibus (GEO) database is a gene expression database that stores chip, second-generation sequencing, and other high-throughput sequencing data. To identify differentially expressed genes (DEGs) in CCA, we applied strict criteria of |log2fold change (FC)| ≥ 1 and an adjusted *p*-value < 0.05 for differential expression analysis of two CCA datasets: GSE107943 and GSE45001 using the online GEO2R tool. GEO2R is a free online tool for DEG analysis developed by the National Center for Biotechnology Information (NCBI) for its GEO database. This tool automatically performs built-in basic correction for batch effects and systematic errors on individual GSE datasets. The GSE107943 dataset contained 57 samples, including 30 cancer tissues and 27 adjacent non-tumorous tissues. The GSE45001 dataset consisted of ten paired cancer and para-cancerous samples. The Gene Expression Profiling Interactive Analysis (GEPIA) platform was utilized to characterize cancer-specific expression genes by comparing transcriptomic data from 36 CCA samples in The Cancer Genome Atlas (TCGA) with nine normal tissues from the Genotype-Tissue Expression (GTEx) project.

To systematically characterize cancer-specific transcriptional signatures, we generated volcano plots for the GSE107943 and GSE45001 datasets via the R package. Venn diagrams were used to visualize the commonly upregulated genes in the GSE107943, GSE45001 and TCGA_GTEx. To evaluate the prognostic significance of the gene of interest in CCA, we utilized the GEPIA platform to generate survival curves comparing overall survival between high- and low-expression groups.

### Clinical samples tissues

Totally, 50 paired pathologically confirmed CCA and adjacent normal tissues collected from Affiliated Hospital of Qingdao University were used in this study. The methods and experimental protocols were approved by the Ethical and Scientific Committees of Affiliated Hospital of Qingdao University. All the enrolled patients obtained informed consent.

### Cell culture

Human CCA cell lines HuCCT1 and RBE, as well as human embryonic kidney cells HEK293T were obtained by Icellbioscience (Shanghai, China). HuCCT1 and RBE cells were cultured in RPMI-1640 medium containing 10% fetal bovine serum (FBS), while HEK293T cells were incubated in DMEM supplemented with 10% FBS. All cells were maintained in a humidified incubator with 5% CO_2_ at 37 °C.

### Lentiviral construction and infection

A pLVX-IRES-puro vector carrying CST1 CDS fragment (NM_001898) driven by the CMV promoter was generated. pLVX-shRNA1 vectors expressing two distinct short hairpin RNAs (shRNAs) targeting CST1 (5’-GAAGAAACAGTTGTGCTCTTT-3’ and 5’-CCAGGCCATTCGCACCAGCCA-3’) were constructed. Using Lipofectamine 3000 (L3000015, Invitrogen, Carlsbad, California, USA), the vector was co-transfected with pSPAX2 packaging vector and pMD2.G envelope vector into HEK293T cells, and concentrated by ultracentrifugation. The generated lentivirus was infected into HuCCT1 and RBE cells, after which maintained in 2 μg/mL puromycin for 14 days to select stable CST1-overexpressing or -silencing clones.

### Cell Counting Kit-8 (CCK-8) assay

In the CCK-8 assay, the water-soluble tetrazolium salt WST-8 [2-(2-methoxy-4-nitrophenyl)-3-(4-nitrophenyl)-5-(2,4-disulfophenyl)-2h-tetrazolium sodium salt] is reduced by intracellular dehydrogenases to generate an orange-yellow formazan dye, which is soluble in tissue culture media. The quantity of formazan produced—reflected by the color intensity—is directly proportional to the number of viable cells. Cell proliferation was assessed using the CCK-8 kit (BS350A, Biosharp Life Sciences, Hefei, China.) following the manufacturer’s instructions. Briefly, cells were seeded into 96-well plates at a density of 3 × 10³ cells per well, with 5 replicate wells set for each group. After 48 h of culture at 37 °C in a 5% CO₂ incubator, 10 μL of CCK-8 reagent was added to each well, and the plates were incubated for an additional 2 h under the same conditions. Optical density (OD) values were then measured at 450 nm using an 800TS microplate reader (BioTek, Winooski, VT, USA).

### 5-Ethynyl-2’-deoxyuridine (EdU) staining

The Biosharp BL915A Click-iT EdU-488 Kit operates by incorporating the thymidine analog EdU into DNA during synthesis, followed by a Click reaction that labels EdU with Alexa Fluor 488, enabling proliferating cells to emit bright green fluorescence under a fluorescence microscope. According to the manufacturer’s protocol, cells were incubated with 10 μM preheated EdU staining solution in a 5% CO₂ incubator at 37 °C for 3 h. Subsequently, cells were fixed with fixation buffer at room temperature for 15 min and washed twice with PBS. After removing PBS, cells were treated with permeation buffer for 15 min at room temperature, and then incubated with the Click reaction cocktail in the dark for 30 min. After removing the reaction solution, cells were counterstained with DAPI for 5 min. Images were captured under a IX53 inverted fluorescence microscope (Olympus, Tokyo, Japan) at 400 times magnification. The EdU-positive rate was calculated as (number of EdU-positive cells/total cell number) × 100%. To evaluate pyrimidine dependency, CST1-depleted HuCCT1 cells were treated with 100 µM thymidine (dThd) for 24 h prior to EdU staining.

### Flow cytometric detection of cell cycle

Upon completion of trypsinization, the enzymatic reaction was terminated by adding complete culture medium. Subsequently, cell suspensions were centrifuged at 150 g for 5 min to pellet the cells. The cell pellets were gently resuspended in pre-chilled 70% ethanol. The cell-ethanol suspensions were then stored at 4 °C overnight to ensure complete fixation. After the fixation period, the cells were washed twice with PBS, and the supernatant was removed. The cell pellets were resuspended in 500 μL of propidium iodide (PI) staining buffer. The suspension was incubated for 30 min at room temperature in the dark to allow DNA binding. Cell cycle distribution was determined using a flow cytometer by quantifying PI-stained DNA content.

### Senescence β-galactosidase staining

For the detection of SA-β-Gal activity, cells were fixed with β-Gal fixative solution (G1580, Solarbio Life Sciences, Beijing, China) for 15 min at room temperature. Following a three-step washing process, the cells were incubated with β-Gal staining solution at 37 °C overnight in the dark. Stained cells were visualized under an optical microscope to document SA-β-Gal-positive cells, and quantitative analysis was performed by counting positive cells across multiple fields using ImageJ software.

### In vitro immunofluorescence (IF) staining

HuCCT1 cells were fixed with 4% paraformaldehyde (15 min) and permeabilized with 0.1% Triton X-100 (30 min) at room temperature. Following blocking with 1% bovine serum albumin (BSA), specimens were incubated overnight at 4 °C with anti-γ-H2AX^S139^ (the histone H2Ax phosphorylated at Ser139; details in Table S1). Subsequently, slides were stained with Cy3-conjugated goat anti-rabbit IgG (1:200 dilution) for 60 min at room temperature. Nuclei were counterstained with 4’,6-diamidino-2-phenylindole (DAPI), and samples were mounted using antifade mounting medium prior to imaging at 400 times magnification under a fluorescence microscope.

### Cell migration and invasion assays

Cells resuspended in 200 μL of serum-free medium were seeded in a Transwell chamber (8 μm pore size; 14341, Macklin, Shanghai, China), while 500 μL of complete medium containing 10% FBS was added to the bottom of 24-well plates to induce cell migration. If the purpose is to measure the cell invasiveness, Matrigel (356234, Corning Costar, Corning, NY, USA) was spread in the Transwell membrane 2 h in advance, and placed in the incubator at 37 °C to solidify. After incubating for 24 h, the culture inserts were fixed with 4% paraformaldehyde for 20 min and stained in 0.5% crystal violet. Cells that stayed on the top of the membrane were gently scraped by a cotton swab. Migrated/invaded cells on the lower surface were photographed and counted under an inverted microscope at 200 times magnification.

### Enzyme-linked immunosorbent assay (ELISA)

Cytokine and protease levels in cell supernatants were quantified using commercially available ELISA kits. Specifically, interleukin-6 (IL-6), C-C motif chemokine ligand 20 (CCL20), matrix metallopeptidase 2 (MMP2), and matrix metallopeptidase 9 (MMP9) were measured according to the manufacturer’s protocols (all purchased from Fine Test, Wuhan, China).

### Real-time quantitative reverse transcription PCR (RT-qPCR)

Total RNA was extracted from tumor samples or CCA cells using Trizol reagent (RP1001, Bioteke, Beijing, China), and reverse-transcribed into cDNA using an All-in-One SuperMix Kit (MD80101, Magen Biotechnology, Guangzhou, China). The RT-qPCR reactions were performed using a 2 × Fast Taq plus PCR Master Mix (10 μL), SYBR Green (0.5 μL), cDNA (1 μL), and primers (1 μL; sequence details in Table S2). Gene expression levels were normalized to β-actin as an internal control, with relative quantification calculated using the 2^-ΔΔCt^ method.

### Western blot analysis

Cells were analyzed using a radioimmune precipitation assay (RIPA) buffer (PR20001, ProteinTech, Rocky Hill, NJ, USA) supplemented with a protease inhibitor cocktail (PR20032, ProteinTech). Cellular protein concentrations were quantified using a bicinchoninic acid (BCA) kit (PK10026, ProteinTech). Equal protein amounts (15-30 μg) of each lysate were used for the sodium dodecyl sulfate-polyacrylamide gel electrophoresis (SDS-PAGE). Proteins were transferred onto polyvinylidene fluoride membranes, which were then blocked with 5% skimmed milk in Tris-buffered saline with Tween 20 (PR20011, ProteinTech) and incubated overnight at 4 °C with primary antibodies (details in Table S1). Afterwards, the membrane was probed with horseradish peroxidase-conjugated with goat anti-mouse or anti-rabbit IgG secondary antibodies (1:10,000 dilution) for 60 min at room temperature. Protein bands were visualized using an ultrasensitive ECL detection kit (PK10003, ProteinTech).

### Statistical analysis

Statistical analysis was performed using GraphPad Prism 8. The experiment was repeated at least three times, and the experimental results were expressed as the mean ± standard deviation (SD). Data that passed normality and lognormality tests were analyzed using the student’s t-test for two-group comparisons, or either a one-way or two-way analysis of variance for multi-group comparisons. Data that failed to pass normality and lognormality tests were analyzed using the Wilcoxon signed-rank test. A p-value < 0.05 was statistically significant.

## Supplementary information

Suplementary information

Full and uncropped western blots

## Data Availability

The datasets used during the current study are available from the corresponding author on reasonable request.
