# Supplementary material for: NFATC2-mediated CST1 upregulation drives cholangiocarcinoma growth and metastasis
Source: Cell Death Discov. 2026 Mar 25;12:187. doi: 10.1038/s41420-026-03036-8 (PMC13139392; doi:10.1038/s41420-026-03036-8)
Supplement: Supplementary file 1 — Suplementary information [file 41420_2026_3036_MOESM1_ESM.docx]

**NFATC2-Mediated CST1 Upregulation Drives Cholangiocarcinoma Growth and Metastasis**

Wei Zhao^1*^, Jing Zhao^2^, Kun Li^1^, Jian Shi^1^, Liyuan Cong^1^, Guangyi Yu^1^

^1^ Department of Hepatobiliary and Pancreatic Surgery, The Affiliated Hospital of Qingdao University, Qingdao, People’s Republic of China.

^2^ Department of Pathology, The Affiliated Hospital of Qingdao University, Qingdao, People’s Republic of China.

^*^ Corresponding author: Dr. Wei Zhao, E-mail: [zhaoweirandy@qdu.edu.cn](mailto:zhaoweirandy@qdu.edu.cn). ORCID: 0000-0003-2549-8063.

**Supplementary methods**

**Animal experiments**

All animal works were performed in accordance with protocols approved by the Animal Experimentation Ethics Committee of Affiliated Hospital of Qingdao University. A pHIV-Luc-ZsGreen lentivirus was infected with HuCCT1 cells stably expressed/silenced CST1, namely Lv-Luc-transduced HuCCT1. For orthotopic liver implantation models, 2×10^6^ Lv-Luc HuCCT1 cells were implanted into the left hepatic lobe of 6-week-old male BALB/c nude mice. After 6 weeks, tumor formation and metastasis were visualized by an *in vivo* bioluminescence system after intraperitoneal injection of D-luciferin (150 mg/kg). For pulmonary metastasis models, 2×10^6^ Lv-Luc HuCCT1 cells were injected into the tail vein of 6-week-old male BALB/c nude mice. At 8 weeks post-injection, lung metastases were assessed via bioluminescence imaging after D-luciferin administration. Upon euthanasia, tumor tissues were resected, photographed, and fixed in 4% paraformaldehyde. After paraffin embedding, serial sections were prepared for immunofluorescence (IF), hematoxylin-eosin (H&E) staining, and immunohistochemical (IHC) examination.

***Ex vivo* double IF staining**

Paraffin-embedded tissues were sectioned at 5 μm, dewaxed, rehydrated, and subjected to heat-mediated antigen retrieval. Following blockage with 1% BSA (15 min, room temperature), slides were simultaneously incubated with paired primary antibodies (details in **Table S1**) for 15 min. After three washes with PBS, specimens were stained with fluorophore-conjugated secondary antibodies [FITC-anti-rabbit IgG (1:200 dilution) and Cy3-anti-mouse IgG (1:200 dilution)] for 90 min at room temperature. Nuclei were counterstained with DAPI, and slides were mounted using antifade mounting medium. Representative images were acquired under a fluorescence microscope at 100 times magnification (Olympus).

***Ex vivo* H&E staining**

Tissue sections (5 μm) were dewaxed, rehydrated, and stained with hematoxylin (H8070, Solarbio) and eosin (A600190, Sangon, Shanghai, China). Sections were subsequently examined under an Olympus BX53 microscope at 40 times magnification.

***Ex vivo* IHC analysis**

Tissue specimens (5 μm) were deparaffinized in xylene and rehydrated through a graded ethanol series. Following heat-mediated antigen retrieval, endogenous peroxidase activity was quenched with 3% hydrogen peroxide. After blocking with 1% BSA, sections were incubated overnight at 4°C with primary antibodies (details in **Table S1**), followed by a 45-min incubation with HRP-conjugated goat anti-rabbit IgG (1:100 dilution) at room temperature. Sections were subsequently developed using diaminobenzidine (C520017, Sangon), counterstained with hematoxylin (H8070, Solarbio), dehydrated, and mounted with neutral balsam. Immunohistochemical staining was visualized under a light microscope at 200 times magnification (Olympus Corporation, Tokyo, Japan).

**Proteomics analysis**

Label-free proteomics analysis was performed by APTBIO Co., Ltd, Shanghai, China. HuCCT1 cells were infected with either CST1-expressing lentiviral vectors or empty vector controls. Total cellular proteins were extracted using RIPA lysis buffer supplemented with protease inhibitor cocktails, resolved via SDS-PAGE, and stained with Coomassie Brilliant Blue R-250 to verify protein integrity. Proteins were digested with trypsin, desalted using C18 Cartridge, and freeze-dried. The obtained peptides were analyzed via LC-MS/MS, using a timsTOF Pro mass spectrometer coupled to an NanoElute HPLC system. Peptides were separated on a C18 analytical column (Thermoscientific EASY column, 25 cm, ID75 μm, 1.9 μm) at a flow rate of 300 nL/min. The mass spectrometer was operated in parallel accumulation–serial fragmentation mode, with a full MS scan (m/z 100-1700). Raw MS data were processed using MaxQuant software and searched against the Homo sapiens UniProt database. Proteins were quantified using label-free quantification (LFQ) based on mass spectral intensity. Principal component analysis (PCA) was performed via to assess inter-group variability and intra-group reproducibility. Differentially expressed proteins (DEPs) were identified based on a |log2FC| >1 and p-value <0.05. Volcano plot, heatmap and Kyoto Encyclopedia of Genes and Genomes (KEGG) pathway enrichment analysis of DEPs were performed via R package software.

**Metabolomics analysis**

Untargeted metabolomics analysis was conducted by Shanghai APTBIO Co., Ltd. HuCCT1 cells were lysed using ice-cold acetonitrile/methanol/H_2_O (2:2:1 v/v/v) mixture. The samples were separated using the Vanquish UHPLC system, and then analyzed by the Thermo Q Exactive mass spectrometer operated in both positive and negative electrospray ionization (ESI) modes. Raw data files were converted to mzXML format using ProteoWizard and processed with XCMS for peak alignment, retention time correction and peak area extraction. Metabolites were then identified using public databases and in-house library. PCA and orthogonal partial least squares-discriminant analysis (OPLS-DA) were performed to visualize group clustering and identify discriminatory metabolites. Differential expressed metabolites (DEMs) were identified based on |log2FC| >1, p-value < 0.05 and variable importance in the projection (VIP) > 1. Volcano plot and KEGG pathway enrichment analysis of DEPs were performed via R package.

**Double luciferase reporter assay**

The CST1 promoter sequence with -2000/+35 or -1000/+35 was inserted into the pGL3-basic vector to construct the CST1 promoter luciferase reporter plasmid. HEK293T cells were co-transfected with the reporter plasmid and either an empty vector or NEATC2 overexpression plasmid using Lipofectamine 3000 (Invitrogen) according to the manufacturer’s protocol. After a 48-h incubation period, the luciferase activity was measured using the Dual-Luciferase Reporter Gene Assay System (KGE3302, KeyGEN, Nanjing, China) and normalized to Renilla luciferase activity as an internal control.

**Chromatin immunoprecipitation polymerase chain reaction (ChIP-PCR)**

The ChIP assay was performed using a commercial kit (P2078, Beyotime Biotech, Shanghai, China) following the manufacturer’s protocol. Briefly, cells were crosslinked with 1% formaldehyde for 10 min at 37°C and sonicated into DNA fragments at 4°C. The sonicated lysate was incubated with anti-NEATC2, anti-H3 (positive control) or anti-IgG (negative control) antibody at 4°C overnight. Protein A+G agarose beads were then added into the lysates and incubated for an additional 1 h at 4°C. The immunoprecipitated DNA was eluted, de-crosslinked, and purified. Purified DNA was amplified by PCR using specific primers targeting CST1 promoter regions 3, 4, and 5 (details in **Table S2**). PCR products were electrophoresed on 2% agarose gels.

**Electrophoretic mobility shift assay (EMSA)**

Nuclear extracts from HuCCT1 cells were prepared using the Nuclear Protein Extraction Kit (EX2550, Solarbio), and protein concentrations were determined by the BCA Protein Assay Kit (PC0020, Solarbio). NFATC2 DNA-binding activity was subsequently assessed using the Chemiluminescent EMSA Kit (SIDET101, Viagene, Jiangsu, China) per the manufacturer's protocol.

**Supplementary figure and tables**

**Fig. S1** **CST1 knockdown suppresses proliferation and metastasis in RBE cells (Related to Fig. 2)**

**(A)** Expression of CST1 in human RBE cells were detected by western blot. **(B)** The number of viable cells after 48 h of incubation was evaluated by the CCK8 assay. **(C, D)** The DNA replication capacity of RBE cells was assessed and quantified by EdU incorporation assay (C, bar = 50 μm), with EdU-positive cells subsequently quantified (D). **(E, F)** Cell cycle distribution of RBE cells was analyzed by flow cytometry (E: upper; F: left). Cellular senescence in RBE cells was assessed by SA-β-gal staining (bar = 100 μm), with positive cells subsequently quantified (E: lower; F: right). **(G, H)** Migration and invasion capabilities of RBE cells were evaluated by Transwell chamber assays with and without Matrigel (bar = 100 μm). **(I)** IL-6 and CCL20 mRNA expression and secreted levels in RBE cells were assessed by RT-qPCR and ELISA, respectively. **(J)** Secretion levels of invasion-related factors MMP2 and MMP9 in RBE cell supernatants were quantified by ELISA. **(K)** Expression of mesenchymal markers N-cadherin and Vimentin in RBE cells were detected by western blot. Data in (B, D, F, H-J) were presented as mean ± SD. SA-β-gal, senescence-associated β-galactosidase. Full and uncropped western blots were shown in Supplemental Material.


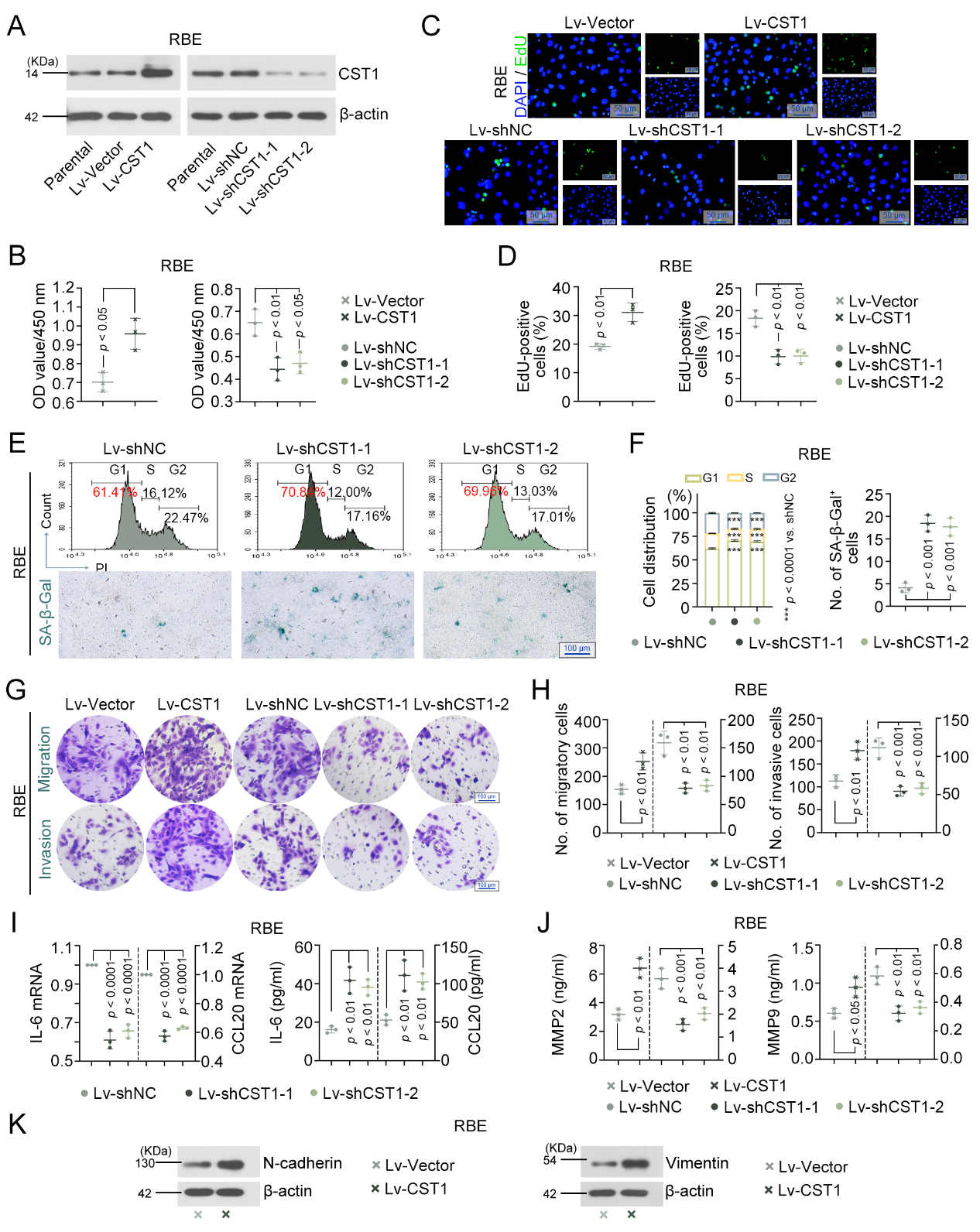


**Fig. S2 CST1 silencing suppresses CCA metastasis *in vivo* (Related to Fig. 3)**

**(A)** Schematic illustration of lung metastasis models inoculated with HuCCT1 cells. **(B)** Representative bioluminescence images of pulmonary metastatic lesions. **(C)** Left, quantitative analysis of metastatic nodule numbers in lungs. Right, representative images of lung tumors and H&E staining for lung metastasis assessment. Data in (C) were presented as mean ± SD.


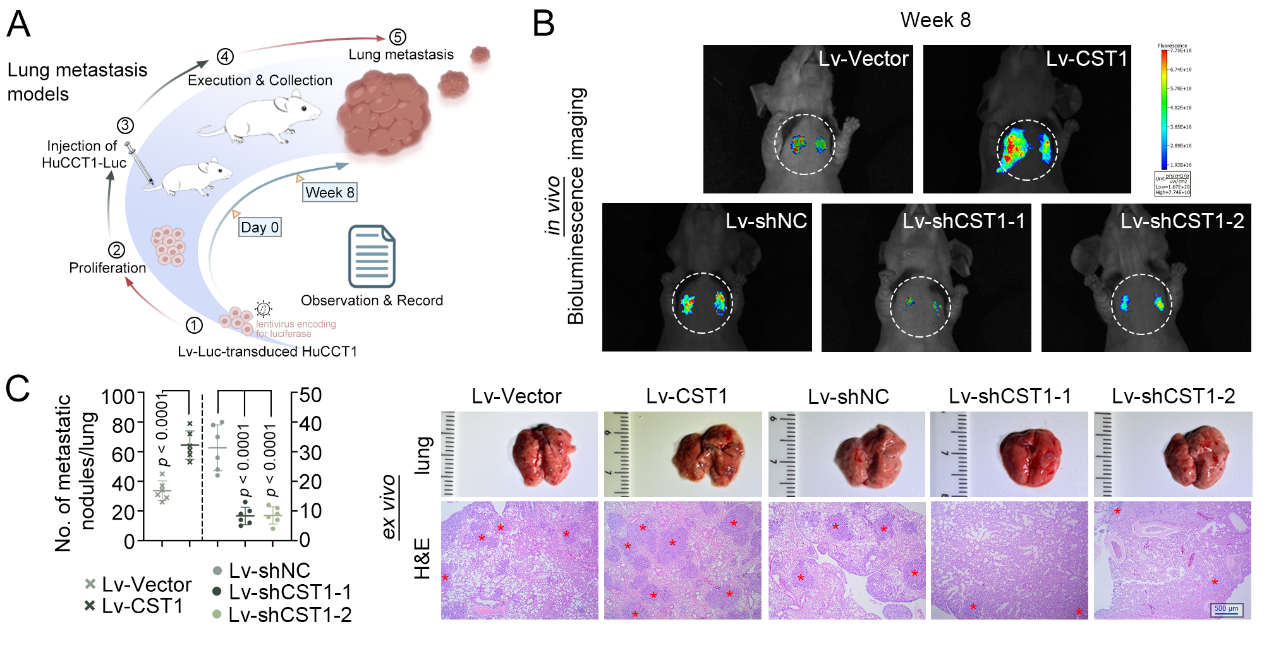


**Fig. S3 NFATC2 activates CST1 transcription**

**(A)** JASPAR database showed putative binding sites of NFATC2 with a score >8 on the CST1 promoter. **(B, C)** mRNA expression levels of NFATC2 and CST1 in HuCCT1 cells were measured by RT-qPCR. **(D)** Protein expression of NFATC2 and CST1 in HuCCT1 cells were detected by western blot. **(E)** Dual-luciferase activities of CST1 promoter reporter vectors in HEK293T cells. **(F)** ChIP-PCR analysis of NFATC2 occupancy at regions 3–5 of the CST1 promoter in HuCCT1 and RBE cells. Input: whole cell lysate; IgG, negative control. **(G)** ChIP-PCR verification of NFATC2 binding to regions 3 (upper), 4 (middle), and 5 (lower) of the CST1 promoter in HuCCT1 cells. Input, whole-cell lysate; H3, positive control; IgG, negative control. **(H)** EMSA analysis of NFATC2 DNA-binding activity at region 3. Data in (B, C, E) were presented as mean ± SD. Full and uncropped western blots were shown in Supplemental Material.


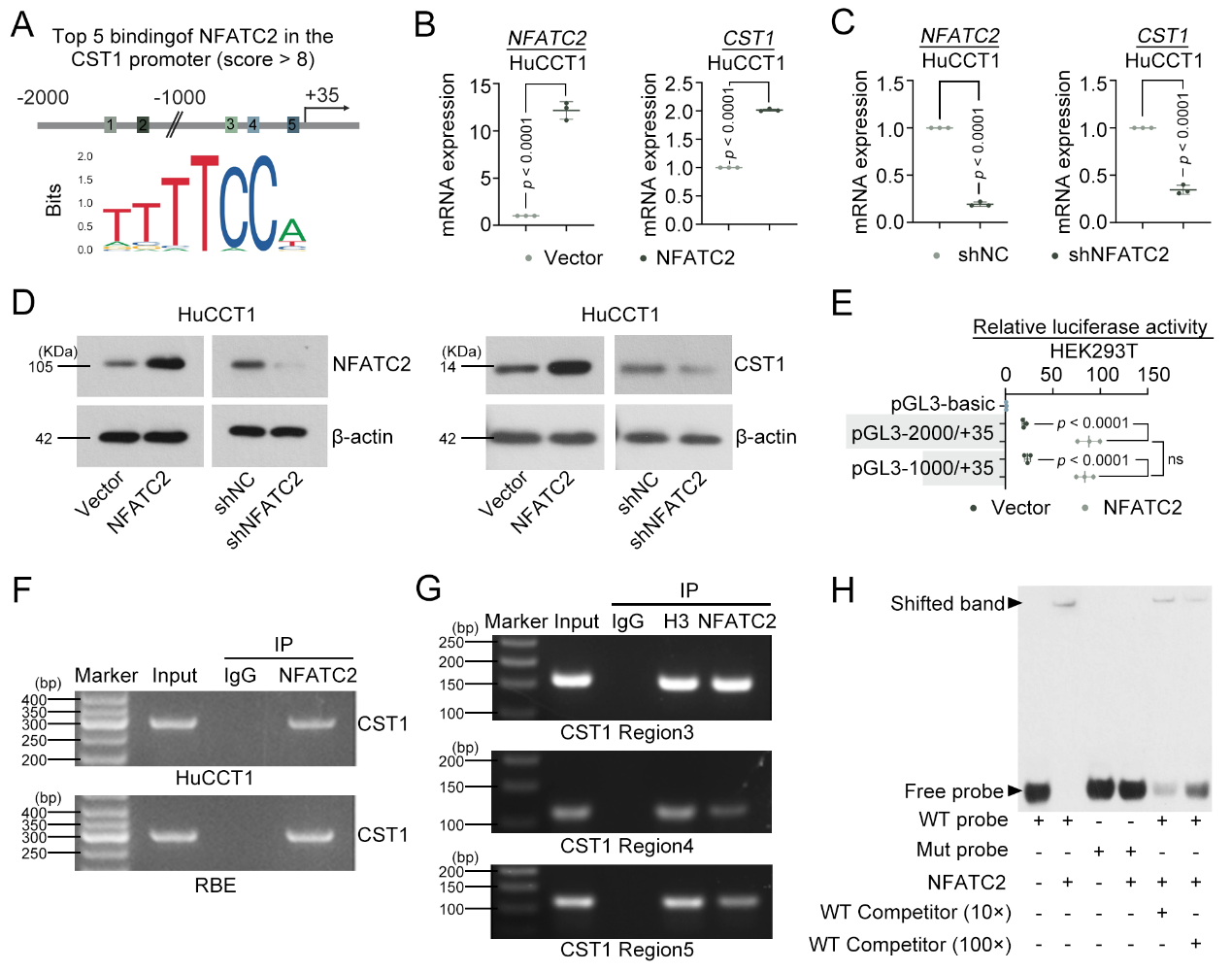


**Fig. S4 NFATC2 functions as an upstream regulator of CST1**

**(A)** Cell viability assessed by CCK-8 assay at 48 h post-incubation. **(B, D)** Cell cycle distribution of HuCCT1 cells was analyzed by flow cytometry. **(C, E)** Cellular senescence evaluated by SA-β-gal staining (C; bar, 100 μm) and quantification of positive cells (E). **(F-H)** Migration and invasion capabilities of HuCCT1 cells were evaluated by Transwell chamber assays with and without Matrigel. Data in (A, B, D, E, G, H) were presented as mean ± SD.


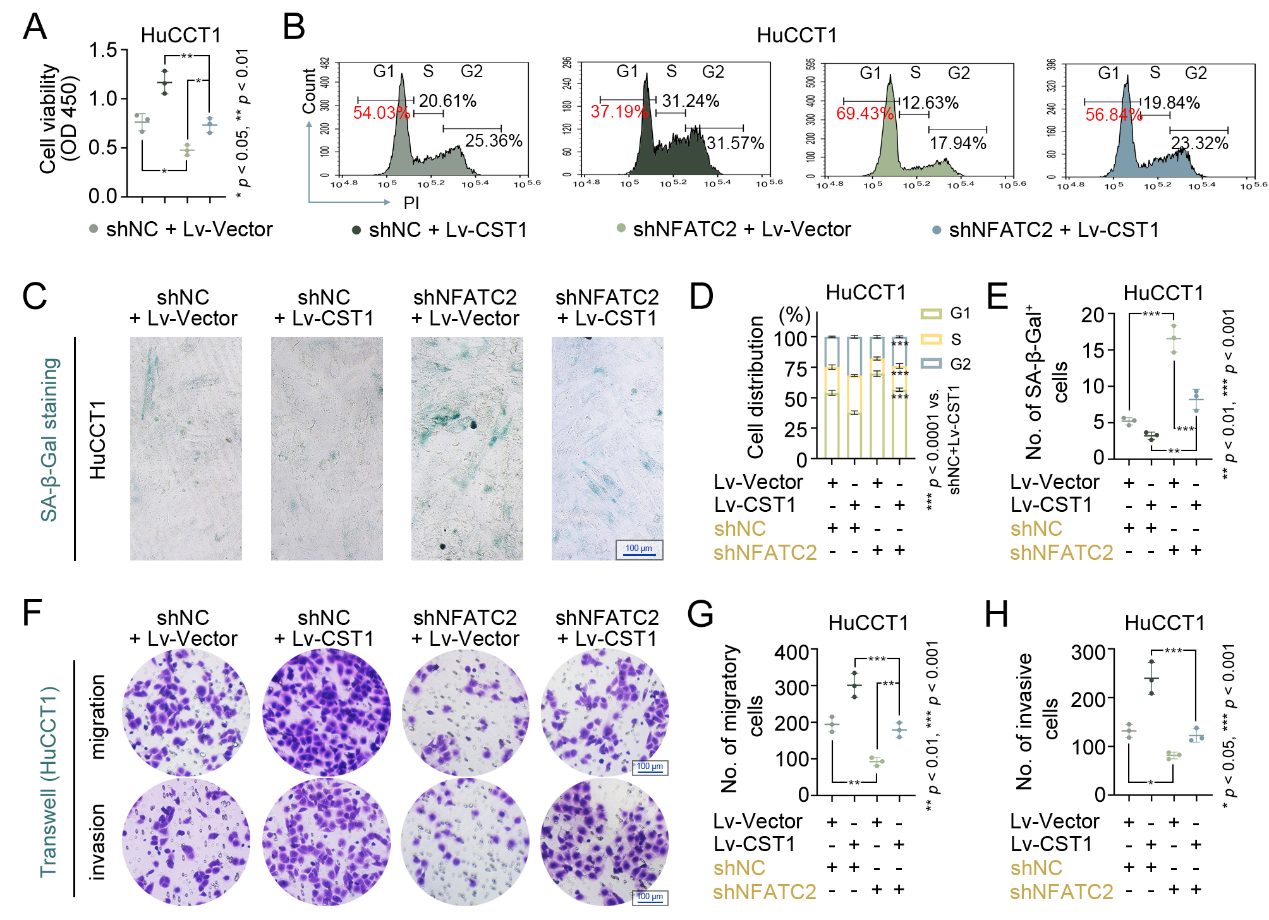


**Tab. S1 The primary antibodies used in western blot, IHC and IF analyses**

| **Purpose** | **Symbol** | **Catalog** | **Company** | **Dilution** |
| --- | --- | --- | --- | --- |
| WB | CST1 | 670423 | Zenbio | 1：500 |
|  | N-cadherin | 22018-1-AP | ProteinTech | 1：1000 |
|  | Vimentin | A19607 | Abclonal | 1：500 |
|  | TYMS | R25907 | Zenbio | 1：500 |
|  | SOX4 | 680786 | Zenbio | 1：500 |
|  | NFATC2 | 251865 | Zenbio | 1：1000 |
|  | β-actin | 66009-1-Ig | ProteinTech | 1：20000 |
| IHC | SOX4 | 680786 | Zenbio | 1:100 |
|  | Ki67 | A20018 | Abclonal | 1:200 |
| IF | cytokeratin 7 | T200296 | Zenbio | 1: 50 |
|  | CST1 | 16025-1-AP | Proteintech | 1:100 |
|  | γ-H2AX^S139^ | R381558 | Zenbio | 1: 50 |

**Tab. S2 The primer sequences for RT-qPCR and ChIP-PCR analyses**

| **Purpose** | **Symbol** | **Sequences (5’-3’)** | |
| --- | --- | --- | --- |
| RT-qPCR | CST1 | Forward | TATCTGAGTACCCTGCTGC |
|  |  | Reverse | TCATTGAGGTCTGCGTTAT |
|  | NFATC2 | Forward | ATCCCTGAATATCGGAACAA |
|  |  | Reverse | TGGGTGGTAGGTAAAGTGC |
|  | CCL20 | Forward | GACCGTATTCTTCATCCTA |
|  |  | Reverse | ATTTCACCCAAGTCTGTTT |
|  | IL-6 | Forward | TTCGGTCCAGTTGCCTTCT |
|  |  | Reverse | GTGCCTCTTTGCTGCTTTC |
| ChIP-PCR | CST1 | Forward | GGGAGTAGCTGGTGATAG |
|  |  | Reverse | GTGACACTGGTCCTTTGA |
|  | CST1 region 3  (-529/-523) | Forward | CTTCAAGCCCTAGACCTCC |
|  |  | Reverse | AAGACCCTGTCCATACCCT |
|  | CST1 region 4  (-434/-428) | Forward | GGCAAAGAAATTCTCCACA |
|  |  | Reverse | CAGGAGCACTTGGTCACAG |
|  | CST1 region 5  (-352/-346) | Forward | CCTGGATGCCCCTGATA |
|  |  | Reverse | GGTGACACTGGTCCTTTGA |
