# Supplementary figures and images for: NFATC2-mediated CST1 upregulation drives cholangiocarcinoma growth and metastasis
Source: Cell Death Discov. 2026 Mar 25;12:187. doi: 10.1038/s41420-026-03036-8 (PMC13139392; doi:10.1038/s41420-026-03036-8)

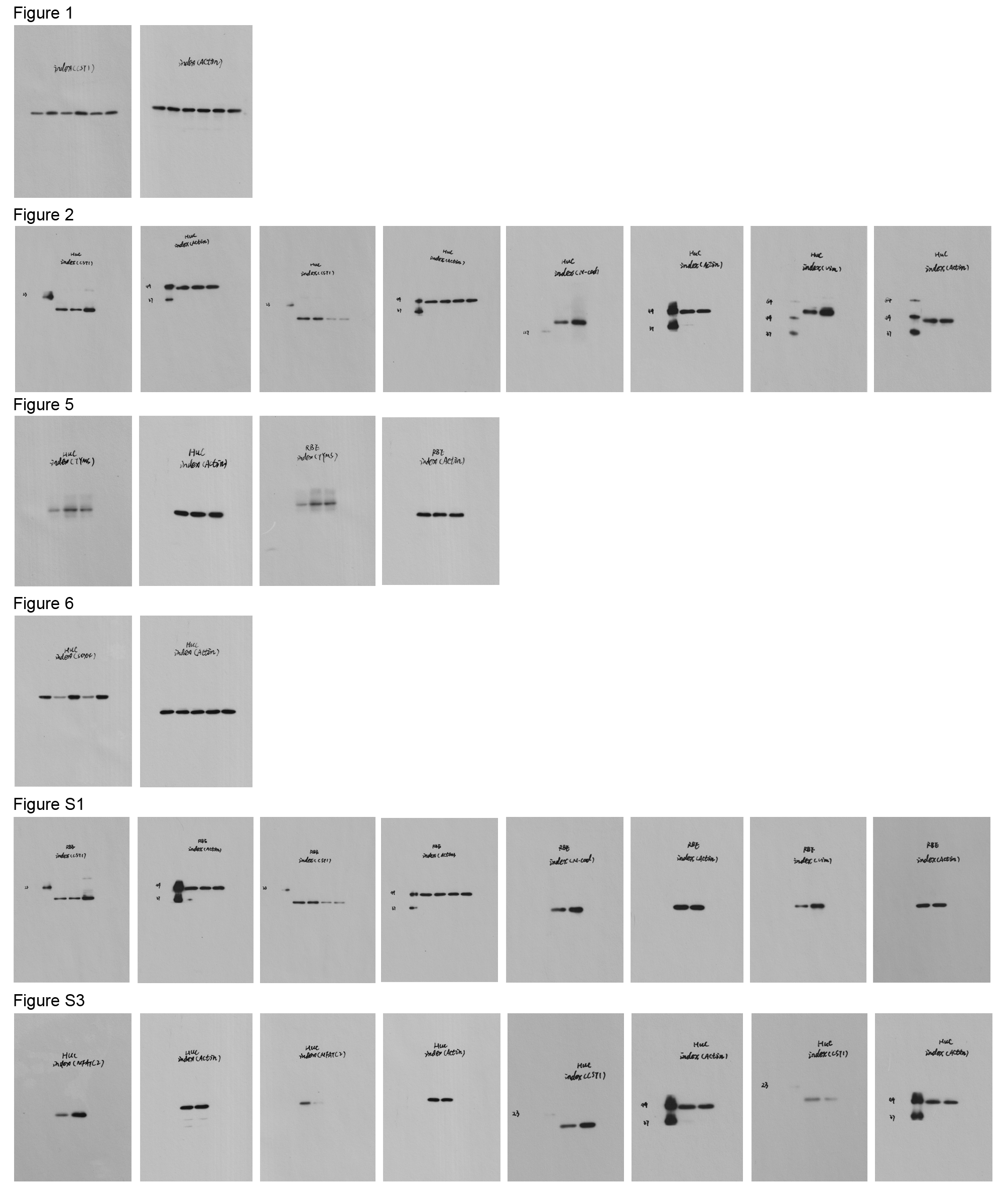

Supplement: Supplementary file 2 — Full and uncropped western blots [file 41420_2026_3036_MOESM2_ESM.png]
